# Development of a population-anchored Z-score for MRI-based knee osteoarthritis disease activity: Data from the Osteoarthritis Initiative

**DOI:** 10.1016/j.ocarto.2026.100848

**Published:** 2026-06-26

**Authors:** Jonggyu Baek, Julieann C. Patarini, Emily Kirillov, Nhung Vo, Michael J. Richard, Ming Zhang, Kate L. Lapane, Matthew S. Harkey, Grace H. Lo, Shao-Hsien Liu, Charles B. Eaton, Jamie MacKay, Timothy E. McAlindon, Jeffrey B. Driban

**Affiliations:** a Department of Population and Quantitative Sciences, UMass Chan Medical School, 55 Lake Avenue North, Worcester, MA, 01655, USA; b Division of Rheumatology, Allergy, & Immunology, Tufts Medical Center, 800 Washington, Street, Boston, MA, 02111, USA; c Department of Computer Science, Boston University, 111 Cummington Mall, Boston, MA, 02215, USA; d Department of Kinesiology, Michigan State University, 308 W. Circle Drive, East Lansing, MI, 48824, USA; e Department of Medicine, Baylor College of Medicine, One Baylor Plaza, Houston, TX, 77030, USA; f Medical Care Line and Research Care Line, Houston VA HSR&D Center for Innovations in Quality, Effectiveness and Safety, Michael E. DeBakey Medical Center, 2002 Holcombe Blvd, Houston, TX, 77030, USA; g Center for Primary Care and Prevention, Kent Hospital, 111 Brewster St, Pawtucket, RI, 02860, USA; h Brown University School of Public Health, 121 South Main Street Providence, RI, 02903, USA; i Department of Radiology, University of Cambridge, Box 218, Cambridge Biomedical Campus, Cambridge, CB2 0QQ, United Kingdom; j Norwich Medical School, University of East Anglia, Norwich Research Park, Norwich, NR4 7TJ, United Kingdom; k Division of Rheumatology, Department of Medicine, UMass Chan Medical School, 55 Lake Avenue North, Worcester, MA 01655, USA

**Keywords:** Disease activity, Composite metric, Z-score

## Abstract

**Objective:**

We previously developed a novel imaging biomarker, reflecting whole-knee bone marrow lesion and effusion-synovitis volumes, to measure disease activity. This study describes the process used to develop a z-score and its percentile to improve interpretability and comparability across studies.

**Design:**

We used data from several Osteoarthritis Initiative projects (n = 1084 participants with 6876 observations). For 76% of observations, participants had radiographic knee osteoarthritis (Kellgren-Lawrence grade (KL) ≥ 2). We targeted a synthetic reference population with an overall prevalence of 25% for radiographic knee osteoarthritis. A Box-Cox transformation was used to derive a standard normal distribution for a composite metric. A within-cluster resampling method was used to summarize the reference population and estimate model parameters. The new z-score was associated with categories of WOMAC knee pain to evaluate construct validity (cross-sectionally and longitudinally).

**Results:**

The probability of being a normal distribution for a Box-Cox transformed composite metric was 99.5%, and the probability that the newly developed disease activity z-score follows the standard normal distribution was 95.7%. Because increasing age was associated with an upward shift in the mean z-score distribution, we also developed an age-adjusted z-score. We found 94% consistency between the new z-score and the initial composite metric of disease activity and construct validity with WOMAC knee pain.

**Conclusion:**

The unadjusted and age-adjusted z-score for disease activity developed using a synthetic reference population improves both the statistical and clinical usefulness of the disease activity metric. This new composite metric improves comparability across studies, clinical interpretability, and statistical modeling utility.

## 1 Introduction

The development of a composite magnetic resonance imaging (MRI) biomarker reflecting whole-knee bone marrow lesion (BML) and effusion-synovitis volumes has provided a promising approach to quantifying knee osteoarthritis (KOA) disease activity (DA). Prior work demonstrated that this composite metric predicts important clinical outcomes, including WOMAC knee pain progression and incident KOA [[1], [2], [3]]. The initial metric was calculated as the sum of six standardized BML volumes and a standardized whole-knee effusion-synovitis volume, both adjusted for knee size [4].

Despite its predictive utility, the original composite metric has several limitations. First, each component was standardized using cohort-specific means and standard deviations, making the scale dependent on the distribution of the source dataset. As a result, values are not directly comparable across studies that use different samples. Second, the composite score lacks a population-referenced interpretation. For example, a value such as 1.7 does not indicate where a patient falls relative to a meaningful reference population, limiting its interpretability. Third, the raw composite metric is right-skewed, which complicates statistical modeling and interpretation of effect sizes in regression analyses.

To address these limitations, we sought to transform the existing composite metric into a reference-calibrated, normally distributed z-score anchored to a synthetic population with an epidemiologically informed prevalence of radiographic KOA. Mapping the composite metric onto a standard normal distribution allows scores to be interpreted as percentiles within a reference distribution, facilitating clinical communication. In addition, a reference-calibrated z-score enhances comparability across studies, as standardized scores derived using fixed parameters are directly transportable. Finally, a normally distributed metric improves statistical modeling by reducing the influence of skewness and providing stable regression estimates.

Our objectives were to: (1) develop a Box-Cox-transformed, reference-calibrated z-score for MRI-based KOA disease activity; (2) construct and characterize a synthetic reference population with a target prevalence of radiographic KOA to anchor the distribution; and (3) evaluate the construct validity of the resulting z-score in relation to categories of WOMAC knee pain (cross-sectionally and longitudinally). To our knowledge, this is the first effort to anchor an MRI-based OA DA metric to a population-calibrated reference distribution. Through this approach, we aimed to enhance the interpretability, cross-study comparability, and methodological robustness of an established imaging-based DA metric.

## 2 Materials and methods

### 2.1 Study samples

We used several Osteoarthritis Initiative (OAI) datasets from various projects (R01 AR076411 (cohort study), U01 FD007002 [case-control study, cases = knee replacement], R01 AR074447 [case-cohort study, cases = incident KOA], R01 AR065977 [case-control study, cases = accelerated KOA], and U01 **AR067168** [two cohort studies]). Because participants could contribute data from both knees and at multiple time points, the pooled dataset included 1851 participants and 11,071 knee-level observations.

We restricted analyses to White and Black/African American participants due to small numbers in other racial groups, resulting in 1811 participants. We also excluded participants missing any individual component variables required for the DA metric (i.e., BML, effusion-synovitis) as these measures were not collected in all contributing projects. The final analytic sample included 1084 participants contributing 6876 knee-level observations.

Because several contributing projects used case-control or enriched sampling designs, the pooled analytic dataset contained a higher prevalence of radiographic KOA than expected in the general population. Overall, 74% observations had radiographic KOA (Kellgren-Lawrence [KL] grade≥2), and 26% observations did not. This enriched distribution from the OAI, which over-represents the prevalence of radiographic KOA, motivated the construction of a synthetic reference population to anchor the DA metric to a more realistic prevalence of radiographic KOA based on population-based studies.

### 2.2 Sampling the synthetic reference population

The objective of the synthetic reference population was not to estimate national prevalence, but to establish a stable reference distribution for standardizing the DA metric. To calibrate this distribution, we selected a target radiographic KOA prevalence of 25%, informed by large U.S. epidemiologic studies. In the Framingham OA study, radiographic KOA prevalence was reported at 27.4% in ages 60–69 years and 34.1% in ages 70–79 years [5]. Similarly, the Johnston County OA project reported the overall prevalence of KOA at 28% and, by age category, at 14.9% in ages 45–54 years, 26.2% in ages 55–64 years, 36.1% in ages 65–74 years, and 49.9% in ages ≥75 years [6]. Additionally, a national representative study, NHANES III (1991–1994), reported 37.4% of radiographic KOA in ages ≥60 years [7]. Given the variability across age groups and study designs, a 25% prevalence was selected as a pragmatic mid-range anchor to approximate U.S. population estimates while avoiding overrepresentation of OA severity from enriched study samples.

To generate the synthetic reference distribution, we implemented a within-cluster resampling approach. Because participants could contribute data from both knees, at multiple visits, and sometimes multiple OAI projects, sampling at the observation level would disproportionately weight individuals with more measurements. Therefore, we sampled unique participants to ensure equal contribution to each synthetic dataset. For each iteration, we randomly selected participants in a 1:3 ratio of those with radiographic KOA (KL ≥ 2) to those without OA (KL = 0 or 1), yielding 80 participants with OA and 241 without OA per resampled dataset. This procedure was repeated 5000 times. Across iterations, we derived the composite DA metric and estimated transformation and standardization parameters. In addition to a 1:3 ratio of KOA and non-KOA, we provided reference parameters of mean and standard deviation by varying a non-KOA ratio from 1 to 6 (50%–14.3% KOA prevalence). Averaging estimates across 5000 resampled datasets reduced sampling variability and provided stable reference parameters for the final z-score calculation [8].

### 2.3 Box-Cox transformation for normality

The composite DA score was derived from six BML volumes (medial femur, lateral femur, medial tibia, lateral tibia, medial patella, and lateral patella) and whole-knee effusion-synovitis volume. To account for differences in knee size, each component was adjusted for bone width prior to summation. Specifically, let $X_{i}$ be an individual component and $BW_{i}$ be an observed bone width for participant i. The bone width adjusted component was calculated as follows: $X_{i}^{BW}=X_{i}*\frac{\mu_{BW}}{BW_{i}}$, where $\mu_{BW}$ is the mean bone width across all the cohorts.

**Fig. 1:**
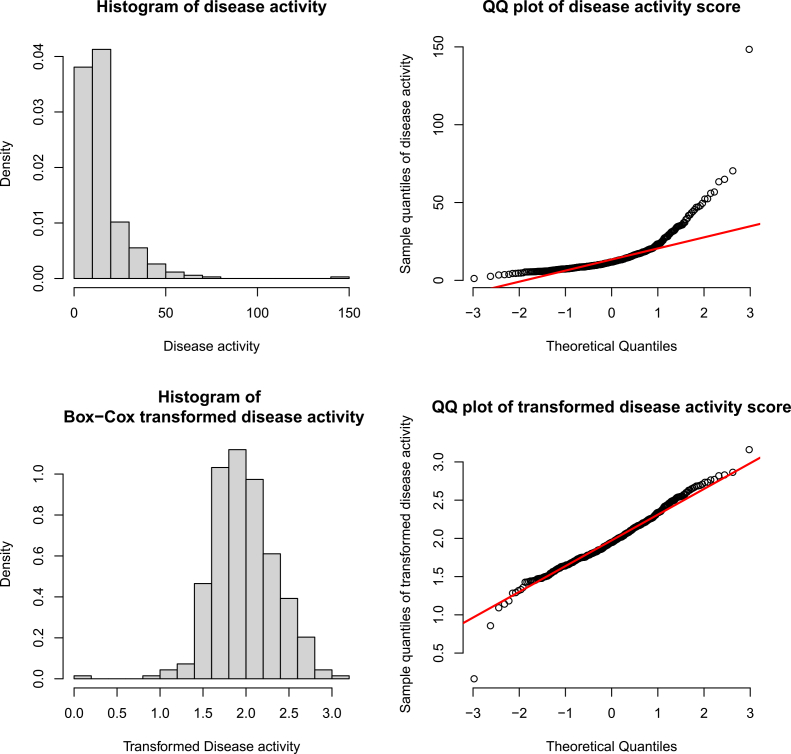
Histogram and Q-Q plot of a raw disease activity (top) and Box-Cox transformed disease activity (bottom).

A raw composite score was calculated by summing the seven adjusted components. Inspection of its empirical distribution revealed substantial right skewness (Fig. 1), limiting interpretability and suitability for parametric modeling. We therefore applied a Box-Cox transformation to approximate normality [9].

The Box-Cox transformation parameter (λ) was estimated within each of the 5000 synthetic reference datasets described above. The optimal λ was determined by averaging the estimated parameters across iterations, yielding λ=−0.2. This value was fixed for subsequent transformation of the composite metric.

Using the pre-specified tuning parameter, $\hat{\lambda }=-0.2$, we transformed the composite score within each resampled dataset and evaluated normality. The proportion of iterations in which the null hypothesis of normality (H_0_: normally distributed vs. H_1_: not normally distributed) was not rejected was calculated across the 5000 datasets to assess the stability of the normal approximation under the synthetic sampling framework.

### 2.4 Estimating reference parameters of a normal distribution for Z-scores

The underlying mean and standard deviation for the Box-Cox transformed DA were unknown. We estimated the reference parameters (mean and standard deviation) with the following procedures: 1) we sampled a dataset with a 1 to 3 ratio of unique participants with and without KOA, 2) we derived the Box-Cox transformed DA and calculated its mean and standard deviation, and 3) the first two steps were iterated 5000 times. Then, the underlying mean ($\mu_{bc-DA}$) and standard deviation ($\sigma_{bc-DA}$) of the Box-Cox transformed DA were estimated by averaging values over 5000 estimates (${\hat{\mu }}_{bc-DA}=1.983$, ${\hat{\sigma }}_{bc-DA}=0.369$). We also repeated the process to estimate the reference mean and standard deviation given a KOA:non-KOA ratio of 1:1 to 1:6 and its corresponding normal distribution test.

The DA z-score was then calculated by standardizing the transformed composite score using the fixed parameters ($\hat{\lambda }=-0.2,{\hat{\mu }}_{bc-DA}=1.983,{\hat{\sigma }}_{bc-DA}=0.369$). In the Results section, we report the proportion of resampled datasets in which the resulting z-scores were consistent with a standard normal distribution (mean = 0, standard deviation = 1) to evaluate the stability and validity of the standardization procedure.

### 2.5 Adjustment and percentile calculation of Z-scores

To evaluate whether demographic or anatomical factors shifted the distribution of DA z-scores, we fitted linear regression models including age, sex, race (Black/African American vs. White), body mass index (BMI) category (underweight/normal, overweight, obese), and knee side (left vs. right). Age was categorized into 10-year intervals (45–54 [reference], 55–64, 65–74, and ≥75 years). The following model was fitted:

$$E\left[DA_{z}|Age,Female,Black,Overweight,Obese,Left\right]$$

$$=\beta_{0}+\beta_{1}Age_{55-64}+\beta_{2}Age_{65-74}+\beta_{3}Age_{75+}+\beta_{4}Female+\beta_{5}Black+\beta_{6}Overweight+\beta_{7}Obese+\beta_{8}Left\text{,}$$

where $\beta_{0}$ is the mean DA z-score for the reference group (White, male, underweight/normal weight participants aged 45–54 years with right-knee measurements), $\beta_{1}$ is the mean z-score difference between age 55–64 years and age 45–54 years, $\beta_{2}$ is the mean z-score difference between age 65–74 years and age 45–54 years, $\beta_{3}$ is the mean z-score difference between age≥75 years and age 45–54 years, $\beta_{4}$ is the mean z-score difference between female and male, $\beta_{5}$ is the mean z-score difference between Black/African American and White, $\beta_{6}$ is the mean z-score difference between overweight and underweight/normal, $\beta_{7}$ is the mean z-score difference between obese and underweight/normal BMI, and $\beta_{8}$ is the mean z-score difference between the left knee and right knee after adjusting for all other variables in the model.

Consistent with the synthetic sampling framework, the regression model was fitted within each of the 5000 resampled datasets (constructed using the 1:3 KOA-to-non-KOA ratio). For each factor, we calculated the proportion of iterations in which the null hypothesis of no association was rejected via stability selection [10]. Factors exceeding a 50% rejection threshold were interpreted as demonstrating consistent evidence of association across the resampling distribution and were therefore retained as adjustment variables and considered to meaningfully shift the z-score distribution [11]. This secondary z-score adjustment may further account for residual demographic or anatomical differences not fully captured by the initial bone-width normalization.

Unadjusted percentiles of DA z-scores were calculated using the standard normal distribution (mean = 0, standard deviation = 1). For adjusted percentiles, covariates identified as shifting the distribution were used to estimate a subgroup-specific mean z-score by summing the model intercept and relevant regression coefficients. Adjusted z-scores were calculated by subtracting this estimated mean shift from the individual's unadjusted z-score, and percentiles were then derived using the standard normal distribution.

An example is provided herein to calculate an adjusted percentile for a Black female participant aged 65–74 years who is overweight with left-knee measures. Assume the fitted regression model resulted in the following estimated coefficients: Intercept ($\beta_{0}$) = −0.2; Age 65–74 ($\beta_{2}$) = 0.30; Female ($\beta_{4}$) = 0.20; Black ($\beta_{5}$) = 0.10; Overweight ($\beta_{6}$) = −0.06; Left knee ($\beta_{6}$) = −0.05, the adjusted mean DA z-score for this group is 0.29 (=−0.2 + 0.3 + 0.2 + 0.1–0.06 - 0.05). If this individual had an observed unadjusted DA z-score of 0.8, the adjusted z-score would be: 0.8–0.29 = 0.51, which corresponds to approximately the 70th percentile of the standard normal distribution.

### 2.6 Radiographic KOA severity

Participants received bilateral weight-bearing, fixed-flexion posterior-anterior knee radiographs. Central readers provided Kellgren-Lawrence grade (KL) grades (0–4) for public release. The KL grades had good agreement between two readings separated by 3–9 months (κ = 0.70 to 0.78; n = 150).

### 2.7 Measuring consistency and construct validity

Using the final analytic sample of 1084 participants contributing 6876 knee-level observations, we evaluated numerical consistency between the newly developed DA z-score and the original composite DA metric. Because the Box-Cox transformation induces a non-linear relationship between the two measures, consistency was assessed using Spearman rank correlation. We additionally evaluated the potential impact of measurement error on the DA z-score by quantifying the expected change in z-score associated with small changes in the component volumes.

To evaluate construct validity, we first examined the distribution of DA z-scores using box plots across WOMAC pain categories: no/minimal pain if WOMAC score≤10, mild if WOMAC score is between 11 and 30, moderate if WOMAC score is between 31 and 50, and severe if WOMAC score>50. We then fitted a linear mixed-effects model to estimate the association between DA z-score and the WOMAC categories. A random intercept for participant was included to account for within-subject correlation arising from bilateral knees and repeated observations over time.

For a quantitative estimate of construct validity, we evaluated the longitudinal association between changes in WOMAC pain and DA z-scores. We used continuous WOMAC pain scores (0–100) and OAI data from baseline to 4 years. Time was coded as years since baseline. For each knee, changes in DA z-scores and WOMAC pain were calculated relative to the first available visit. The relationship between these changes was visualized using bivariate scatterplots with locally weighted scatterplot smoothing. Then, a longitudinal analysis of DA z-scores was performed in a linear mixed-effects model over time, including WOMAC scores as a predictor and an interaction effect between time and WOMAC score. The interaction term is included to evaluate whether the change in DA z-score is different by WOMAC score.

## 3 Result

### 3.1 Synthetic reference population characteristics

The synthetic reference population characteristics were summarized by averaging across all randomly sampled datasets (Table 1). The synthetic population had an average age of 63 years (SD = 9). Fifty-five percent were women, and 12% were Black/African American. The mean BMI was 28.6 kg/m^2^ (SD = 4.2). For KL grade, 29% had KL = 0, 46% had KL = 1, 11% had KL = 2, 9% had KL = 3, and 5% had KL = 4. The prevalence of radiographic KOA by age group was 16% in ages 45–54 years, 20% in ages 55–64 years, 34% in ages 65–74 years, and 33% in ages ≥75 years.

**Table 1:** Average summary statistics averaged (grand mean) across 5000 randomly sampled datasets with a 1 to 3 ratio of unique participants with knee osteoarthritis (OA) (n = 80) or without knee OA (n = 241).

Variable	Label	Mean (SD) or %
Age, years		63.3 (9.2)
Age category, years	≥45 and < 55	20.7
	≥55 and < 65	34.3
	≥65 and < 75	30.7
	≥75+	14.2
Female		55.1
Black		12.4
Body mass index (BMI) (kg/m^2^)		28.6 (4.2)
BMI category	25 ≤ BMI <30 kg/m^2^	42.7
	BMI ≥30 kg/m^2^	35.5
Side of knee	Left	51.6
Kellgren-Lawrence (KL) grade	0	29.3
	1	45.7
	2	11.3
	3	8.7
	4	5.0
KOA (KL ≥ 2) by age category	≥45 and < 55	15.9
	≥55 and < 65	19.5
	≥65 and < 75	33.8
	≥75+	32.8
	
**Individual components of disease activity**^a^		
Effusion-synovitis volume (cm^3^)		11.86 (7.53)
Bone marrow lesion volume – Lateral femur (cm^3^)		1.10 (2.93)
Bone marrow lesion volume – Lateral patella (cm^3^)		0.18 (0.47)
Bone marrow lesion volume – Lateral tibia (cm^3^)		0.79 (2.35)
Bone marrow lesion volume – Medial femur (cm^3^)		0.68 (2.04)
Bone marrow lesion volume – Medial patella (cm^3^)		0.25 (0.53)
Bone marrow lesion volume – Medial tibia (cm^3^)		1.13 (3.20)

a Each component of disease activity is bone width adjusted by calculating as follows: for each component $X_{i}$ from subject i, the bone width ($BW_{i}$) adjusted $X_{i}^{BW}=X_{i}*\frac{\mu_{BW}}{BW_{i}}$, where $\mu_{BW}$ is the mean bone width across all the cohorts.

### 3.2 Normality of Box-Cox transformation and Z-score

Across the 5000 resampled synthetic reference datasets, the null hypothesis of normality for the Box-Cox–transformed DA metric was not rejected in 99.5% of iterations. Hence, the Box-Cox transformation (λ = −0.2) consistently produced an approximately normal distribution of the composite DA score (Fig. 1).

Using the fixed reference parameters ($\hat{\lambda }=-0.2,{\hat{\mu }}_{bc-DA}=1.983$, ${\hat{\sigma }}_{bc-DA}=0.369$), we then evaluated whether the resulting DA z-scores followed a normal distribution. For each resampled dataset, z-scores were calculated using the fixed transformation and reference parameters. Normality was assessed relative to a distribution with mean 0 and standard deviation 1. The null hypothesis of standard normality was not rejected in 95.7% of iterations, indicating stable performance of the standardization procedure under repeated sampling.

Sensitivity analysis of the reference parameters to the assumed KOA prevalence was performed by varying KOA prevalence from 14.3% to 50% (Supplemental Table 1). Across these scenarios, the estimated reference mean, ${\hat{\mu }}_{bc-DA}$, ranged from 1.950 to 2.069, the standard deviation, ${\hat{\sigma }}_{bc-DA}$, ranged from 0.358 to 0.399, and the empirical p-value for normality ranged from 0.989 to 0.997.

### 3.3 Age adjustment

Table 2 presents the mean differences in DA z-scores associated with potential adjustment factors across the resampled datasets. Based on the predefined criterion of an empirical rejection rate exceeding 50%, age was the only factor that meaningfully shifted the distribution of z-scores. Age categories were subsequently included in the final adjustment model to estimate subgroup-specific mean shifts in z-scores. Table 3 presents the empirical rejection rates and corresponding adjustment values for each age category.

**Table 2:** Estimated difference and empirical rejection rate of age category, race, gender, and knee side in association with disease activity z-scores.

Variable	DA z-score	
	Estimate	Empirical rejection rate^b^
Intercept	−0.292^a^	0.226
Age (55–64 vs 45–54 years)	0.335	0.494
Age (65–74 vs 45–54 years)	0.365	**0.574**
Age (≥75 vs 45–54 years)	0.527	**0.788**
Female (vs. Male)	−0.051	0.008
Black/African American (vs. White)	0.208	0.138
BMI category (overweight vs. underweight/normal)	−0.059	0.006
BMI category (obese vs. underweight/normal)	0.049	0.004
Left knee (vs. Right knee)	−0.008	0.009

Bold = empirical rejection rate >0.50, indicating DA z-score needs to be adjusted with that factor.

a The mean disease activity z-score is −0.301 for the right knees of male OAI participants aged 45–54 years who are White from a linear model with disease activity z-score as the dependent variable and all terms included on the rows as predictors.

b Empirical rejection rate is defined as the average number of rejecting each predictor from randomly sampled 5000 data sets, where each data set contains a 1 to 3 ratio of randomly sampled unique participants with knee osteoarthritis (n = 80) and without knee osteoarthritis (n = 241).

**Table 3:** The estimated difference and empirical rejection rate of selected variables in association with disease activity z-scores.

Variable	DA z-score	
	Estimate	Empirical rejection rate^b^
Intercept	−0.299^a^	**0.632**
Age (55–64 vs 45–54 years)	0.339	**0.519**
Age (65–74 vs 45–54 years)	0.344	**0.522**
Age (≥75 vs 45–54 years)	0.497	**0.734**

Bold = empirical rejection rate >0.50, indicating DA z-score needs to be adjusted with that factor.

a The mean disease activity z-score is −0.299 for OAI participants aged 45–54 years from a linear model with disease activity z-score as the dependent variable and all terms included on the rows as predictors.

b Empirical rejection rate is defined as the average number of rejecting each predictor from randomly sampled 5000 data sets, where each data set contains a 1 to 3 ratio of randomly sampled unique participants with knee osteoarthritis (n = 80) and without knee osteoarthritis (n = 241).

The estimated mean shift for the reference group (ages 45–54 years) was −0.299. Relative to this group, the mean DA z-score was higher by 0.339 for individuals aged 55–64 years, 0.344 for those aged 65–74 years, and 0.497 for those aged ≥75 years. These values represent age-specific shifts in the reference distribution and were used to calculate age-adjusted z-scores and percentiles.

### 3.4 Unadjusted and adjusted percentiles

Unadjusted percentiles of DA z-scores were calculated using the standard normal distribution (mean = 0, standard deviation = 1). For example, z-scores of −1, 0, and 1 correspond to the 16th, 50th, and 84th percentiles, respectively.

Age-adjusted percentiles were calculated by incorporating the estimated age-specific shifts in the reference distribution (Table 3). For each age category, the percentile was derived from a normal distribution with a mean equal to the subgroup-specific estimated mean shift and a standard deviation of 1. Specifically, using the 45–54 age group (mean = −0.299) as the reference, means for older participants were derived by applying age-specific beta coefficients. For example, the mean of 0.045 (=−0.299 + 0.344) applies to people ages 65–74 years. Hence, for a 68-year-old individual with an unadjusted DA z-score of 1.7, the percentile was calculated relative to a normal distribution with a mean = 0.045 and standard deviation = 1. This corresponded to the 95.1st percentile (upper 4.9%), indicating higher DA relative to age-matched reference values.

### 3.5 User-friendly web-based interactive application with measurement errors

We developed an interactive web application using the R Shiny tool (https://jongguri.shinyapps.io/score-calculation/) to calculate the DA z-score and its percentile [12]. Once a clinician or investigator inputs a person's sum score, bone width, age, and the potential measurement error (±e), the app will indicate the person's placement (age unadjusted and age adjusted) with the margin of measurement errors based on the reference distribution (Supplementary Fig. 1).

### 3.6 Consistency between new z-scores and original measurements

**Fig. 2:**
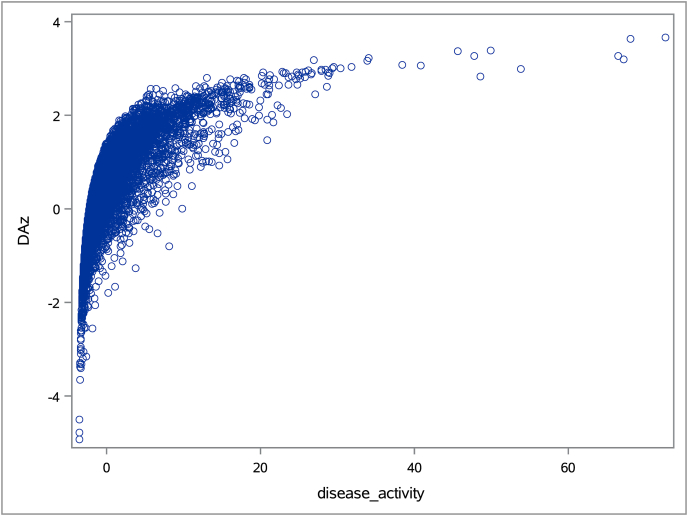
A scatter plot of unadjusted DA z-score (y-axis) and DA.

Fig. 2 illustrates the relationship between the unadjusted DA z-score (y-axis) and the original composite DA metric (x-axis). Consistency between the new z-score and the original composite metric was supported by a strong monotonic agreement between the two measures, based on a rank correlation coefficient of 0.94.

We evaluated the impact of potential measurement error on the DA z-score by deriving the expected change in DA z-scores associated with small perturbations in the original component measures. Details of the derivation are provided in the Supplementary Material. This theoretical analysis demonstrated that the effect of measurement error on the DA z-score depends on the underlying DA value because of the non-linear Box–Cox transformation. Across the observed range of DA values, a 10% and 20% change in DA corresponded to an approximate shift of ±0.08 and ± 0.16 standard deviation in the DA z-score, respectively.

### 3.7 Relationship between z-score and WOMAC pain scores

**Fig. 3:**
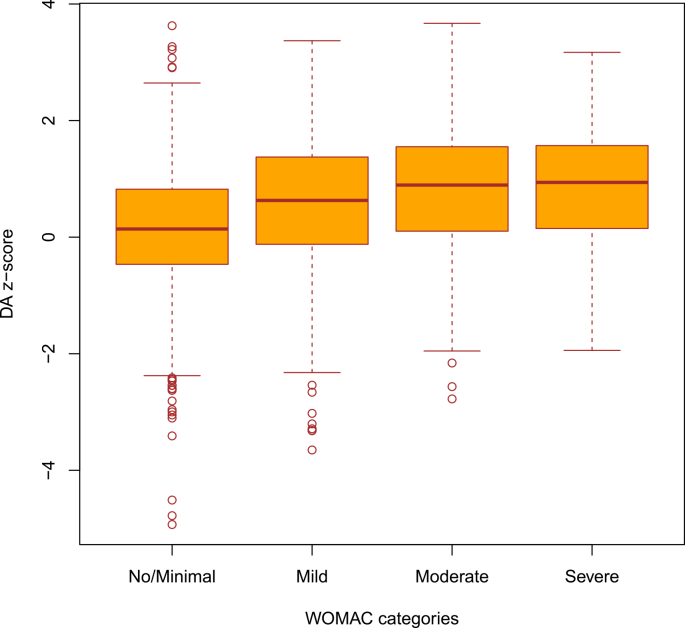
Box plots of DA z-score (y-axis) by WOMAC categories (x-axis).

Fig. 3 illustrates the distribution of DA z-scores across WOMAC pain categories in the final analytic sample. A clear positive trend was observed, with higher WOMAC pain corresponding to higher DA z-scores. Model estimates from the linear mixed-effects model are presented in Table 4. The results demonstrated a statistically significant increase in mean DA z-score with increasing WOMAC pain.

**Table 4:** Estimated cross-sectional association between disease activity (DA) z-score and WOMAC categories from a linear mixed effects model.

Variable	Estimate	SE	95% CI		P value	Comparison	Mean DA	95% CI		P value
			2.50%	97.50%			Difference	2.50%	97.50%	
Intercept	0.228^a^	0.029	0.17	0.285	<0.001					
Mild vs. No/minimal	0.343	0.019	0.305	0.381	0.001	Mild vs. No/minimal	0.343	0.305	0.381	<0.001
Moderate vs. No/minimal	0.518	0.028	0.462	0.573	<0.001	Moderate vs. Mild	0.175	0.122	0.228	<0.001
Severe vs. No/minimal	0.68	0.044	0.594	0.766	<0.001	Severe vs. Moderate	0.162	0.075	0.249	<0.001

Footnote: SE = standard error CI=Confidence interval.

The following linear mixed effects model was fitted for j observation from subject $E\left[DAz_{ij}|WOMAC\;categories\right]=\beta_{0}+b_{0i}+\beta_{1}I\left(WOMAC\;mild\right)+\beta_{2}I\left(WOMAC\;moderate\right)+\beta_{3}I\left(WOMAC\;severe\right)$.

a The mean disease activity z-score is 0.228 for OAI participants with WOMAC categories = no/minimal pain with disease activity z-score as the dependent variable and all terms included on the rows as predictors. Data include all 1084 participants with 6882 observations.

**Fig. 4:**
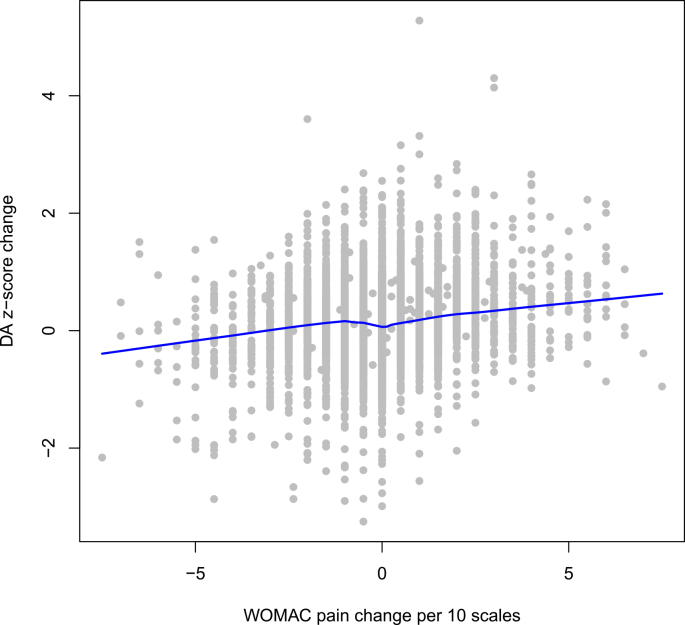
Association between WOMAC change and DA z-score change with empirical locally weighted scatterplot smoothing trend.

For the longitudinal association between changes in WOMAC pain and changes in DA z-score, there is a modest positive linear association (Fig. 4). Overall, greater increases in WOMAC pain are associated with slight increases in DA z-scores, although the relationship appears weak and highly variable. Table 5 shows a longitudinal analysis of DA z-scores over time. The interaction term was not significant (p-value = 0.538), implying that WOMAC pain does not modify the change in DA z-score. The significant effect of time (estimate = 0.099) indicates that DA z-score increases ∼0.1 over time (p-value<0.001). The significant effect of WOMAC pain (estimate = 0.154) indicates that the average DA z-score increases by 0.154 per 10-point increase in WOMAC pain at time 0 (p-value <0.001), and this increase is consistent across visits. Considering a 20-point increase in WOMAC pain as the minimal clinically important difference [13], the result may imply that a ∼0.3 point increase in DA z-score would indicate clinically significant change.

**Table 5:** Longitudinal association between disease activity (DA) z-score and WOMAC scores from a linear mixed effects model.

Variable	Estimate	SE	95% CI		P value
			2.50%	97.50%	
Intercept	−0.014^a^	0.035	−0.083	0.055	0.687
Time	0.099	0.009	0.082	0.115	<0.001
WOMAC score per 10 increase	0.154	0.009	0.136	0.172	<0.001
Interaction between time and WOMAC score per 10 increase	−0.002	0.003	−0.009	0.005	0.538

Footnote: SE = standard error CI=Confidence interval.

The following linear mixed effects model was fitted for j observation from subject $E\left[DAz_{ij}|WOMAC\;scores\right]=\beta_{0}+b_{0i}+\beta_{1}time_{ij}+\beta_{2}WOMAC_{ij}+\beta_{3}time_{ij}*WOMAC_{ij}$, where WOMAC is scaled by 10 increment.

a The mean disease activity z-score is −0.014 for OAI participants with WOMAC score 0 (no pain) at time = 0 (the baseline visit) with disease activity z-score as the dependent variable and all terms included on the rows as predictors. Data include all 1084 participants with 6882 observations.

## 4 Discussion

MRI (MRI) based quantification of BML and effusion-synovitis volumes has emerged as a promising approach for measuring KOA DA. However, the existing composite metric is inherently cohort-dependent, lacks a population-referenced interpretation, and exhibits right-skewness that complicates statistical modeling. These limitations restrict its interpretability, cross-study comparability, and distributional stability. In this study, we address these challenges by transforming the original composite metric into a population-referenced, transportable z-score anchored to an epidemiologically informed reference distribution. By calibrating the metric to a synthetic population and normalizing its distribution, we provide a standardized measure that enables meaningful percentile interpretation, facilitates comparability across studies, and improves statistical robustness for clinical and research applications.

This developed z-score has important implications. A percentile-based z-score allows clinicians to communicate DA to their patients in intuitive ways (e.g., “your knee DA is higher than 90% of people at your age”), potentially improving clinician-patient communication and shared decision making. Because this metric is anchored to a stable reference distribution, it enables longitudinal monitoring, allowing changes over time to be interpreted relative to a fixed reference population benchmark.

Age was associated with shifting the distribution of DA z-score. When a clinician pinpoints a person's percentile based on the reference distribution, the percentile can be calculated after adjusting the mean distribution shift for their age category so that DA can be interpreted relative to age-matched peers. The unadjusted z-score remains appropriate for statistical regression modeling when age is included as a covariate or a predictor variable. Thus, age adjustment refines interpretability for percentile-based communication, whereas the unadjusted z-score preserves analytic flexibility for etiologic and prognostic modeling.

The developed DA z-score imposes equal weighting when summing the 7 individual components for the composite score, but the influence of each variable on the composite score may not be equal due to correlation between the components. After adjusting the bone width, effusion-synovitis could have a numerically larger volume than the BMLs, implying that effusion-synovitis would have a larger influence on the composite score. However, the large influence of effusion-synovitis could be partly offset by the inclusion of six BML components. Although the equal weighting approach offers transparency, simplicity, and interpretability, a previous study examined other weighting approaches and found that they offer no major improvement in performance [1]. Given the absence of clear performance gains, the equal-weighting strategy was retained to preserve interpretability and comparability.

We acknowledge that we used various projects originally designed for specific outcomes. Our goal was to develop an MRI-based DA z-score with participants from these projects, because the BML and effusion-synovitis volumes were consistently measured across all projects. The uniform application of imaging and segmentation methods across projects should minimize differential measurement bias in developing the composite z-score. Therefore, the development of the z-score is unlikely to be substantially affected by selection bias.

Several limitations warrant consideration. First, the synthetic reference population was derived from the OAI and is not nationally representative. Although we calibrated the reference distribution to a target 25% prevalence of radiographic KOA based on prior epidemiologic studies, this anchor was a pragmatic choice rather than a definitive population benchmark. However, we presented alternative parameter calibrations by varying the KOA prevalence (Supplementary Table 1), which changed the estimated mean and standard deviation of the Box-Cox transformed DA metric only modestly (mean: 2.069–1.950; SD: 0.399–0.358). Hence, the resulting DA z-score is relatively robust to reasonable assumptions regarding the prevalence of radiographic KOA in the reference population. Second, our analytic sample was restricted to White and Black/African American participants due to small numbers in other racial and ethnic groups, which may limit generalizability to more diverse populations. Third, the Box–Cox transformation parameter and reference standardization parameters were estimated using OAI data. While the within-cluster resampling approach improves stability, external validation in independent cohorts is needed to assess transportability. Future studies should examine associations with additional imaging, biochemical, and patient-reported outcomes to further establish validity across disease domains.

In summary, this study introduces a reference-calibrated z-score for MRI-based KOA DA anchored to a target 25% radiographic OA prevalence, enhancing both statistical performance and interpretability. By transforming a cohort-dependent composite metric into a population-referenced measure, this framework improves cross-study comparability, supports more stable statistical modeling, and provides clinicians with an intuitive percentile-based tool for communicating DA and monitoring progression. Furthermore, the standardization in the current study could facilitate pooled analyses across cohorts, improve harmonization in multicenter studies, and support the use of consistent imaging biomarkers in clinical trials.

## Author contributions

All authors declare meeting the authorship criteria as outlined by the ICMJE. Each author has made significant contributions to the work.

Jonggyu Baek made substantial contributions to the conception and design of the work, analysis, and interpretation of data for the work, and the drafting and critical review of the work for important intellectual content.

Julieann C. Patarini made substantial contributions to the acquisition of data for the work, and the drafting and critical review of the work for important intellectual content.

Emily Kirillov made substantial contributions to the acquisition of data for the work and to the critical review of the work for important intellectual content.

Nhung Vo made substantial contributions to the acquisition of data for the work and to the critical review of the work for important intellectual content.

Michael J. Richard made substantial contributions to the acquisition of data for the work and to the critical review of the work for important intellectual content.

Ming Zhang made substantial contributions to the conception and design of the work, the acquisition and interpretation of data for the work, and the critical review of the work for important intellectual content.

Kate L. Lapane made substantial contributions to the conception and design of the work, the interpretation of data for the work, and the critical review of the work for important intellectual content.

Matthew S. Harkey made substantial contributions to the conception and design of the work, the acquisition and interpretation of data for the work, and the critical review of the work for important intellectual content.

Grace H. Lo made substantial contributions to the conception and design of the work and interpretation of data for the work, and the critical review of the work for important intellectual content.

Shao-Hsien Liu made substantial contributions to the conception and design of the work and interpretation of data for the work, and the critical review of the work for important intellectual content.

Charles B Eaton made substantial contributions to the conception and design of the work, the acquisition and interpretation of data for the work, and the critical review of the work for important intellectual content.

Jamie MacKay made substantial contributions to the conception and design of the work, the acquisition and interpretation of data for the work, and the critical review of the work for important intellectual content.

Timothy E. McAlindon made substantial contributions to the conception and design of the work, the acquisition, and interpretation of data for the work, and the drafting and critical review of the work for important intellectual content.

Jeffrey B. Driban made substantial contributions to the conception and design of the work, the acquisition, analysis, and interpretation of data for the work, and the drafting and critical review of the work for important intellectual content.

All authors have approved the final version of the manuscript and agree to be accountable for all aspects of the work.

## Role of funding sources

These analyses were financially supported by a grant from the National Institute of Arthritis and Musculoskeletal and Skin Diseases of the National Institutes of Health under Award Numbers R01-AR076411 and R24-AR085006. The OAI is a public-private partnership comprised of five contracts (N01-AR-2-2258; N01-AR-2-2259; N01-AR-2-2260; N01-AR-2-2261; N01-AR-2-2262) funded by the National Institutes of Health, a branch of the Department of Health and Human Services, and conducted by the OAI Study Investigators. Private funding partners include Merck Research Laboratories; Novartis Pharmaceuticals Corporation; GlaxoSmithKline; and Pfizer, Inc. Private sector funding for the OAI is managed by the Foundation for the National Institutes of Health. This manuscript was prepared using an OAI public use data set and does not necessarily reflect the opinions or views of the OAI investigators, the NIH, or the private funding partners. This work was supported in part with resources at the VA's Health Services Research and Development Service Center for Innovations in Quality, Effectiveness, and Safety (#CIN 13–413) at the Michael E. DeBakey VA Medical Center, Houston, TX. The views expressed in this article are those of the authors and do not necessarily represent the views of the Department of Veterans Affairs. The funding sources had no role in study design; in the collection, analysis, and interpretation of data; in the writing of the report; nor in the decision to submit the article for publication.

## Conflict of interest

Jeffrey B. Driban declares being on the Journal of Rheumatology Editorial Board. Timothy E. McAlindon declares he is a consultant for Sanofi, Kolon TissueGene, Medidata, Organogenesis, and is the owner of Ambulomics and Arthometrics. Jeffrey B. Driban and Timothy E. McAlindon hold a patent for Objective Assessment of Joint Damage, US-20220202356, 2020. Matthew S. Harkey declares being a member of OARSI Board. All authors received funding from NIH/NIAMS R01 AR076411. Jeffrey Driban, Timothy McAlindon, Kate Lapane, Shao-Hsien Liu, Julieann Patarini, and Grace Lo received funding from NIH/NIAMS R24 AR085006.
