# Supplementary material for: Development of a population-anchored Z-score for MRI-based knee osteoarthritis disease activity: Data from the Osteoarthritis Initiative
Source: Osteoarthr Cartil Open. 2026 Jun 26;8(3):100848. doi: 10.1016/j.ocarto.2026.100848 (PMC13352054; doi:10.1016/j.ocarto.2026.100848)
Supplement: Multimedia component 1 [file mmc1.docx]

**Supplementary Table 1. Reference parameters for mean** $\boldsymbol{\mu}_{\boldsymbol{bc-DA}}$ **and standard deviation** $\boldsymbol{\sigma}_{\boldsymbol{bc-DA}}$ **by varying non-KOA ratio to KOA with pre-specified Box-Cox tuning parameter,** $\hat{\boldsymbol{\lambda}}\boldsymbol{=-0.2}$**.**

| Non-KOA ratio | N samples | Mean | SD | P-value* |
| --- | --- | --- | --- | --- |
| 1 | 242 | 2.069 | 0.399 | 0.989 |
| 2 | 121 | 2.012 | 0.381 | 0.9968 |
| 3 | 81 | 1.983 | 0.369 | 0.996 |
| 4 | 61 | 1.964 | 0.366 | 0.9952 |
| 5 | 48 | 1.956 | 0.366 | 0.9904 |
| 6 | 40 | 1.950 | 0.358 | 0.9918 |
| *P-value for testing normal distribution. | | |  |  |

**Supplementary Figure 1. R shiny App to calculate DA z-score and its percentile with age unadjusted and adjusted.**

**
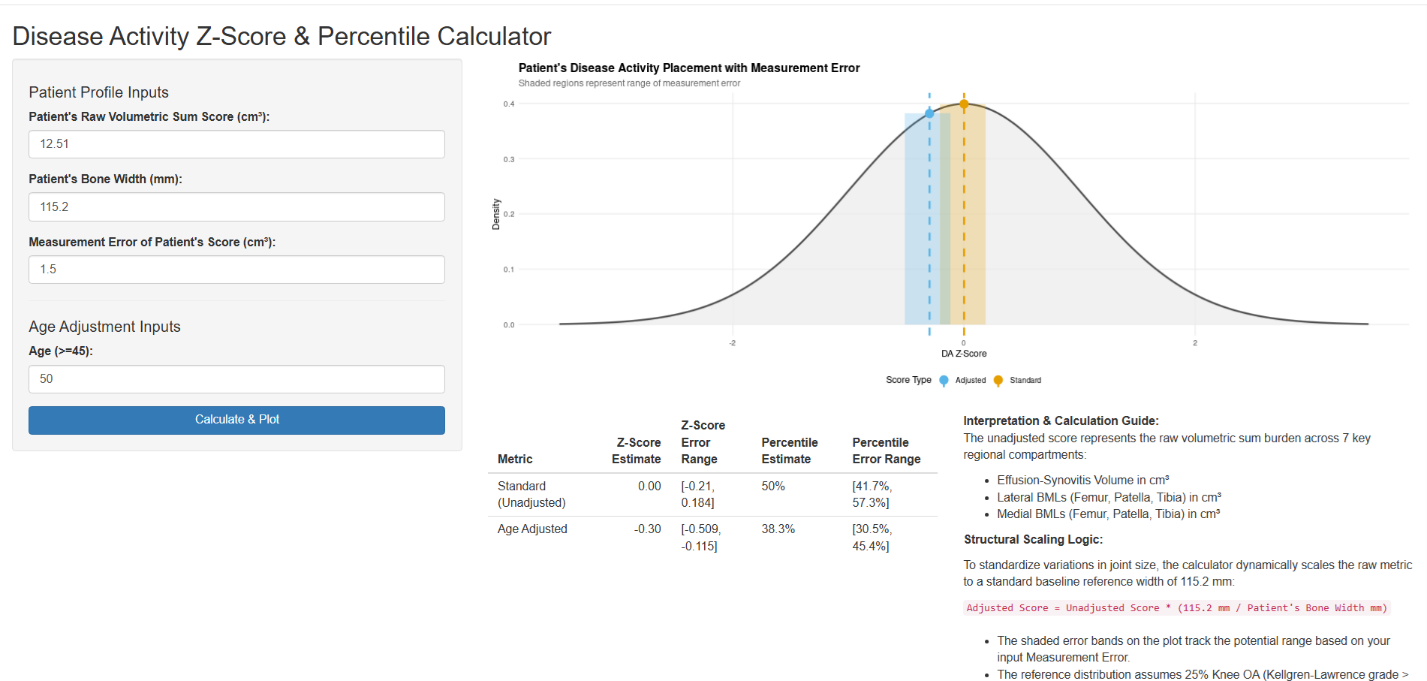
**

**Supplementary Methods: Derivation of DA z-score Measurement Error Resulting from Perturbations in the DA Score**

The DA z-score is constructed through a non-linear transformation of the DA score via a Box-Cox transformation followed by standardization, implying that variability in the underlying DA score does not translate linearly to variability in the DA z-score. The DA score itself is a composite of six BML lesion volume measures and whole-knee effusion-synovitis volume. Therefore, perturbations in the DA score were implemented as proportional perturbations of all underlying component lesion volumes. To characterize this relationship, we conducted a theoretical error propagation analysis based on the Box-Cox transformation and the z-score definition, as detailed below.

The DA z-score was defined as

$$DA_{z} =\frac{BC\left( DA \right)-\hat{\mu}_{bc-DA}}{\hat{\sigma}_{bc-DA}},$$

where BC(DA) denotes the Box-Cox transformed DA score with the estimated Box-Cox parameter ($\hat{\lambda}=-0.2$), $BC\left( DA \right)=\frac{DA^{-0.2}-1}{-0.2}$, the mean $\hat{\mu}_{bc-DA}$=1.983, and the standard deviation $\hat{\sigma}_{bc-DA}$**=0.369.**

For a small perturbation in the DA score (i.e., $\Delta DA$), the corresponding change in the DA z-score ($\Delta DAz$) was approximated as follows:

$$\Delta DAz\approx\frac{DA^{\hat{\lambda}-1}}{\hat{\sigma}_{bc-DA}}*\Delta DA$$

Thus, the expected change in DA z-score depends on both the magnitude of the perturbation and the underlying DA score. Assuming a proportional perturbation $p$, such that $\Delta DA=p*DA$, the resulting change in DA z-score is $\Delta DAz\approx p*\frac{DA^{\hat{\lambda}}}{\hat{\sigma}_{bc-DA}}$, where $\hat{\lambda}=-0.2$ and $\hat{\sigma}_{bc-DA}=0.369$.

Using representative DA values observed in the study population, a 10% perturbation ($p$=0.10) produced an estimated DA z-score change of approximately 0.08 unit, while a 20% perturbation ($p$ = 0.20) provided an estimated change of approximately 0.16 unit.
